# Sequence, Structural and Expression Divergence of Duplicate Genes in the Bovine Genome

**DOI:** 10.1371/journal.pone.0102868

**Published:** 2014-07-23

**Authors:** Xiaoping Liao, Hua Bao, Yan Meng, Graham Plastow, Stephen Moore, Paul Stothard

**Affiliations:** 1 Department of Agricultural, Food and Nutritional Science, University of Alberta, Edmonton, AB, Canada; 2 Queensland Alliance for Agriculture and Food Innovation, The University of Queensland, St Lucia, QLD, Australia; CSIRO, Australia

## Abstract

Gene duplication is a widespread phenomenon in genome evolution, and it has been proposed to serve as an engine of evolutionary innovation. In the present study, we performed the first comprehensive analysis of duplicate genes in the bovine genome. A total of 3131 putative duplicated gene pairs were identified, including 712 cattle-specific duplicate gene pairs unevenly distributed across the genome, which are significantly enriched for specific biological functions including immunity, growth, digestion, reproduction, embryonic development, inflammatory response, and defense response to bacterium. Around 97.1% (87.8%) of (cattle-specific) duplicate gene pairs were found to have distinct exon-intron structures. Analysis of gene expression by RNA-Seq and sequence divergence (synonymous or non-synonymous) revealed that expression divergence is correlated with sequence divergence, as has been previously observed in other species. This analysis also led to the identification of a subset of cattle-specific duplicate gene pairs exhibiting very high expression divergence. Interestingly, further investigation revealed a significant relationship between structural and expression divergence while controlling for the effect of synonymous sequence divergence. Together these results provide further insight into duplicate gene sequence and expression divergence in cattle, and their potential contributions to phenotypic divergence.

## Introduction

Gene duplication is thought to be a major driving force of evolution as it provides raw materials that selection can act upon [1]–[3]. Duplicate genes, the products of gene duplication, initially have identical sequences and functions but tend to diverge in sequence and expression patterns later [4], [5]. The redundancy conferred by duplicate genes may be important for organismal adaptation to different environments [6]. Several previous studies have investigated divergence between duplicate genes at the genome scale [7]–[16]. In *Arabidopsis*, over 95% of duplicate genes studied have diverged in exon-intron structure, and structural divergence occurs largely proportionally to evolutionary time [7]. Gu *et al* examined expression divergence between 400 duplicate gene pairs in yeast using microarray data and found a positive correlation between synonymous sequence divergence (a proxy of evolutionary time) and expression divergence [10]. A similar conclusion was reached in an analysis of human duplicate genes [13]. However, a later study in *Arabidopsis* found that synonymous sequence divergence and expression divergence were not correlated [14].

Duplicate genes are common in eukaryotic genomes and may be responsible for species-specific gene functions, which in turn might facilitate species-specific adaptation [17]–[22]. In farm animals, there is economically important variation in a diverse range of phenotypes related to, for example, reproduction and body structure [23], [24]. In cattle, both natural and artificial selection over a relatively short period of time have resulted in a broad variety of phenotypic and genetically diverse breeds [25]–[27]. Segmental duplications are widespread in the bovine genome and it has been observed that genes overlapping with segmental duplications are significantly enriched for biological functions such as immunity, digestion, lactation and reproduction [26], [28]. However, because of the way segmental duplication is defined, duplicate genes within segmental duplications have high levels of sequence identity [28]. Hence, only duplicate gene pairs with high sequence identity (median = 98.9%) have been investigated previously in cattle. In addition, the relationship between sequence divergence and expression divergence has not been investigated in this species, nor the relationship between gene structure divergence and expression divergence. Investigation of these relationships may provide further insight into the evolution of genes arising through duplication.

The purpose of the present study is to identify duplicate genes in cattle and to characterize their sequence, gene structure and expression divergence.

We found 3131 putative duplicated gene pairs across the genome, including 712 cattle-specific duplicate gene pairs. Cattle-specific duplicate genes are significantly enriched for biological functions such as immunity, growth, digestion, reproduction, embryonic development, inflammatory response, and defense response to bacterium. 3035 (625 cattle-specific) duplicate gene pairs were found to have distinct exon-intron structure, and further investigation showed that exon-intron structure divergence occurred quickly at the early stages of duplicate gene evolution. Expression divergence analysis revealed a positive correlation between synonymous sequence divergence and expression divergence, as expected, and led to the identification of a subset of cattle genes exhibiting high expression divergence. Finally, we investigated the relationship between structural and expression divergence and found that expression divergence was on average greater for duplicate gene pairs exhibiting exon-intron structure divergence, regardless of their overall level of synonymous sequence divergence, suggesting structural divergence may be partially responsible for the divergent expression pattern observed.

## Materials and Methods

### Identification of duplicated gene pairs

All bovine protein sequences were downloaded from Ensembl database release 67 [29]. To identify duplicate gene pairs, a previously described approach was followed [30]. BLASTP [31] was used to compare every protein against all other proteins. Reciprocal best BLAST hits were classified as duplicates if (1) the length of the BLASTP aligned region was ≥80% of the longer protein, and (2) the identity between them was 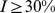 if the aligned region is longer than 150 amino acids and 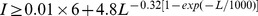 for all the other protein pairs. Pseudogenes were identified based on Ensembl annotation and discarded. Annotations from the Ensembl database were also used to further classify duplicates as non-cattle-specific duplicates (i.e. also observed in other species in the Ensembl database) or cattle-specific duplicates (i.e. observed only in the bovine genome).

### Synonymous and nonsynonymous sequence divergence

Protein sequences for each duplicated gene pair were aligned using ClustalW [32]. The protein alignments were then used as a guide to align the coding sequences (from Ensembl release 67) using a custom Perl script. The synonymous (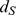) and nonsynonymous (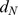) sequence divergence were calculated for each pair of aligned coding sequences, using the maximum likelihood method implemented in the Phylogenetic Analysis Using Maximum Likelihood (PAML) package (version 4) [33].

### Exon-intron structure difference between duplicate gene pairs

A previously described method was followed to determine whether duplicate genes have diverged in exon-intron structure [7]. Duplicate genes were regarded as structurally divergent if they had a different number of exons (termed Class 1 difference) or if they had the same number of exons but the lengths of at least one pair of homologous exons were different (termed Class 2 difference). The number of exons and the length of exons were retrieved from Ensembl database release 67.

### Sample collection, library construction, sequencing and expression profiling

To measure gene expression, mRNA from seven different tissues was extracted (adipose, muscle, hypothalamus, duodenum, liver, lung and kidney) from frozen tissues using TRIzol (Invitrogen). The original samples were collected from beef cattle at the Lacombe research station in Alberta (Canada), following the guidelines of the Canadian Council on Animal Care (1993), and the protocol approved by the Lacombe Research Centre Animal Care Committee. Messenger RNA from 7∼14 animals was pooled equally before sequencing (Table S1). Sequencing libraries were constructed from each pooled tissue according to a standard protocol (mRNA Sequencing Sample Preparation Guide, Illumina, USA).

Sequencing was performed on the Illumina Genome Analyzer II following the manufacturer’s recommendations. Raw reads that failed the chastity filter and reads with average quality less than 20 were removed. The remaining reads were aligned to the UMD3 bovine genome assembly [34] using TopHat v1.4.0 [35]. Cufflinks v1.3.0 [36] was then used to quantify the expression of each transcript in each tissue. Raw sequence data are available in the ArrayExpress database (www.ebi.ac.uk/arrayexpress) under accession number E-MTAB-2596.

### Expression profile similarity between duplicate genes

Following a previous study [37], the FPKM values were log-transformed using log2 (FPKM+offset) with an offset = 1.0. To measure the similarity in expression profile between duplicate genes, Pearson’s correlation coefficient r of expression level across all seven tissues between each duplicate pair was calculated. A high r indicates a high similarity in expression profile between duplicate genes. Furthermore, to study the relationship between 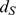 (or 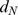) and the correlation coefficient r, we analyzed only the gene pairs in which both genes were expressed in at least one tissue. A gene was treated as not expressed if FPKM was <1 and classified as expressed if FPKM >2. The Pearson’s correlation coefficient r was transformed into 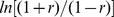. As shown in Figure S1, the transformation serves to make the scale more appropriate for linear regression analysis [10], [13]. The linear regression was carried out between 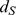 (or 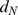) and the transformed r.

## Results

### Distribution of duplicate gene pairs in the bovine genome

**Figure 1:**
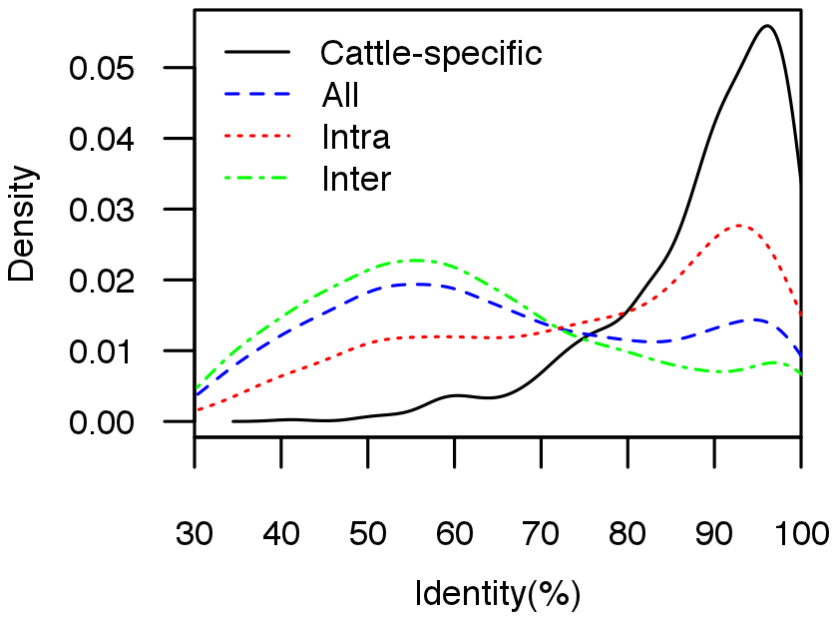
Distribution of sequence identity between duplicate gene pairs.

A total of 3131 putative duplicate gene pairs were identified, including 712 cattle-specific duplicate gene pairs (Table S2). The sequence identities between duplicate gene pairs vary from 30% to 100% (Figure 1). Not surprisingly, the distribution of identities between cattle-specific duplicate gene pairs is greatly shifted to the right when compared with the distribution of all duplicate genes (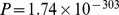, Figure 1). Of 3131 duplicate gene pairs, 2165 (69%) are interchromosomal duplications (Table 1). However, only 215 (30%) cattle-specific duplicate gene pairs are interchromosomal duplications, presumably reflecting the fact that duplicates tend to arise by intrachromosomal duplications but that over time additional changes to the genome can further separate duplicates. Consistent with this, compared with interchromosomal duplications, intrachromosomal duplication gene pairs show obvious higher identities (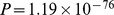, Figure 1), as was also noted in a cattle study of segmental duplications [28]. 712 cattle-specific duplicate gene pairs are distributed in a nonrandom fashion in the genome. Duplicate content varies significantly among different chromosomes (Table 1). Chromosomes 15, 29, and X show the greatest enrichment for duplicate genes with more than twofold the duplicate content of the genome average. Interestingly, Chromosomes 15, 29 and X all have significantly more intrachromosomal duplications than interchromosomal duplications (Table 1).

**Table 1:** Summary of duplicate genes identified.

Chromosome	Number ofgenes inEnsembl	Intrachromosomalduplicategenes	Interchromosomalduplicategenes	Number ofduplicategenes	Cattle-specificinterchromosomalduplicate genes	Cattle-specificintrachromosomalduplicate genes	Number of cattle-specificduplicate genes
1	985	52	175	227(23.0%)	14	24	38(3.9%)
2	1,021	34	201	235(23.0%)	16	18	34(3.3%)
3	1,372	124	258	382(27.8%)	66	23	89(6.5%)
4	855	78	146	224(26.2%)	42	14	56(6.5%)
5	1,323	122	256	378(28.6%)	78	28	106(8.0%)
6	692	28	128	156(22.5%)	8	8	16(2.3%)
7	1,396	144	252	396(28.4%)	64	16	80(5.7%)
8	829	48	135	183(22.1%)	28	6	34(4.1%)
9	602	28	107	135(22.4%)	10	18	28(4.7%)
10	1,074	114	189	303(28.2%)	72	19	91(8.5%)
11	1,047	54	205	259(24.7%)	12	13	25(2.4%)
12	414	12	90	102(24.6%)	4	6	10(2.4%)
13	850	40	178	218(25.6%)	22	18	40(4.7%)
14	571	14	115	129(22.6%)	4	16	20(3.5%)
15	1,050	220	139	359(34.2%)	144	27	171(16.3%)
16	710	36	129	165(23.2%)	12	15	27(3.8%)
17	665	44	119	163(24.5%)	14	17	31(4.7%)
18	1,236	138	167	305(24.7%)	60	12	72(5.8%)
19	1,347	102	230	332(24.6%)	40	15	55(4.1%)
20	384	14	69	83(21.6%)	2	8	10(2.6%)
21	731	22	118	140(19.2%)	18	13	31(4.2%)
22	608	14	127	141(23.2%)	2	11	13(2.1%)
23	785	100	94	194(24.7%)	56	13	69(8.8%)
24	347	18	59	77(22.2%)	6	4	10(2.9%)
25	766	50	116	166(21.7%)	14	5	19(2.5%)
26	437	26	81	107(24.5%)	6	13	19(4.3%)
27	274	16	56	72(26.3%)	10	6	16(5.8%)
28	355	10	61	71(20.0%)	6	5	11(3.1%)
29	705	94	111	205(29.1%)	64	14	78(11.1%)
X	1,128	156	199	355(31.5%)	100	25	125(11.1%)
All	24,559	1952	4310	6262(25.5%)	994	430	1424(5.8%).

### Functional roles of cattle-specific duplicate genes

Consistent with similar duplication studies in the other mammals and a previous cattle segmental duplication study [28], [38]–[41], we also observed many duplicate genes that are important for drug detoxification, immunity and receptor and signal recognition (such as cytochrome P450, ribonuclease A and beta defensins). In order to test whether any particular function is overrepresented in cattle-specific duplicate genes, we performed singular enrichment analysis using agriGO [42]. Statistically significant over representations were observed for multiple biological processes (Table S3), such as ‘response to stimulus’ (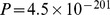), ‘response to chemical stimulus’ (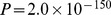), ‘reproduction’ (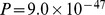), ‘defense response’ (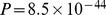), ‘immune system process’ (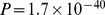), ‘immune response’ (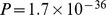), ‘embryonic development’ (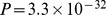), ‘inflammatory response’ (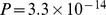), ‘response to wounding’ (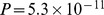), ‘innate immune response’ (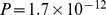), ‘defense response to bacterium’ (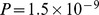), and ‘digestion’ (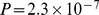). We also observed over representations for several cell components, such as ‘plasma membrane’ (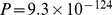), ‘MHC protein complex’ (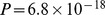) and ‘MHC class I protein complex’ (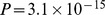).

### Extensive changes in exon-intron structure between duplicate gene pairs

**Figure 2:**
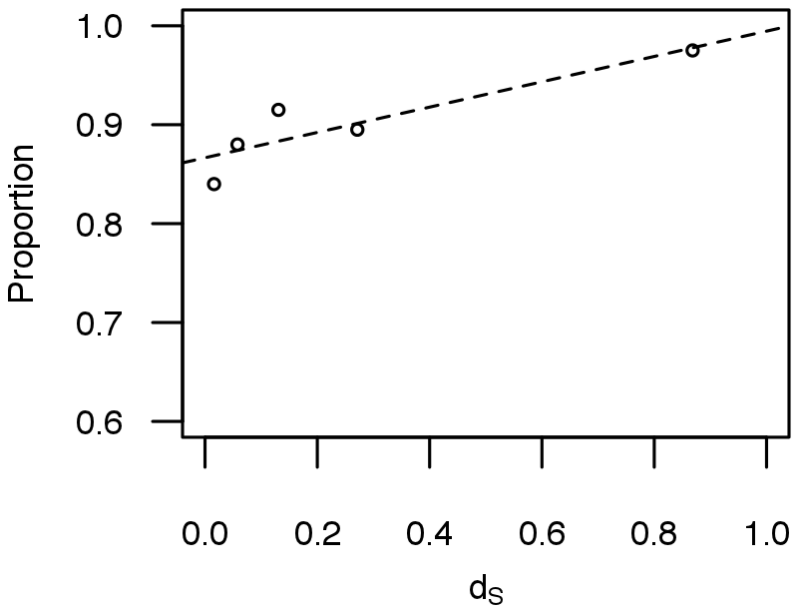
The relationship between proportion of duplicate gene pairs with different exon-intron structure and sequence divergence. Synonymous divergence is used to represent sequence divergence. Each point represents 200 gene pairs.

Comparisons of 3131 duplicate gene pairs showed that 1827 pairs (58.4%) had a different number of exons (Class 1). In 1198 other cases (38.2%), the number of exons remained the same, whereas the lengths of one or more homologous exons were different (Class 2). Our approach of comparing exon numbers and lengths identified 3025 (96.6%) duplicate genes with obvious differences in gene structure. Slightly fewer cattle-specific duplicate gene pairs (87.8% of cattle-specific duplicates, 383 pairs for Class 1 and 242 pairs for Class 2) have different exon-intron structures. We next examined whether the proportion of duplicate gene pairs with different exon-intron structure is correlated with evolutionary time. A significant positive correlation is observed between the proportions of gene pairs with diverged exon-intron structure and 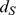 (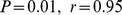, Figure 2), as was also noted in a previous study in Arabidopsis [7].

### Expression divergence of duplicate gene pairs

**Figure 3:**
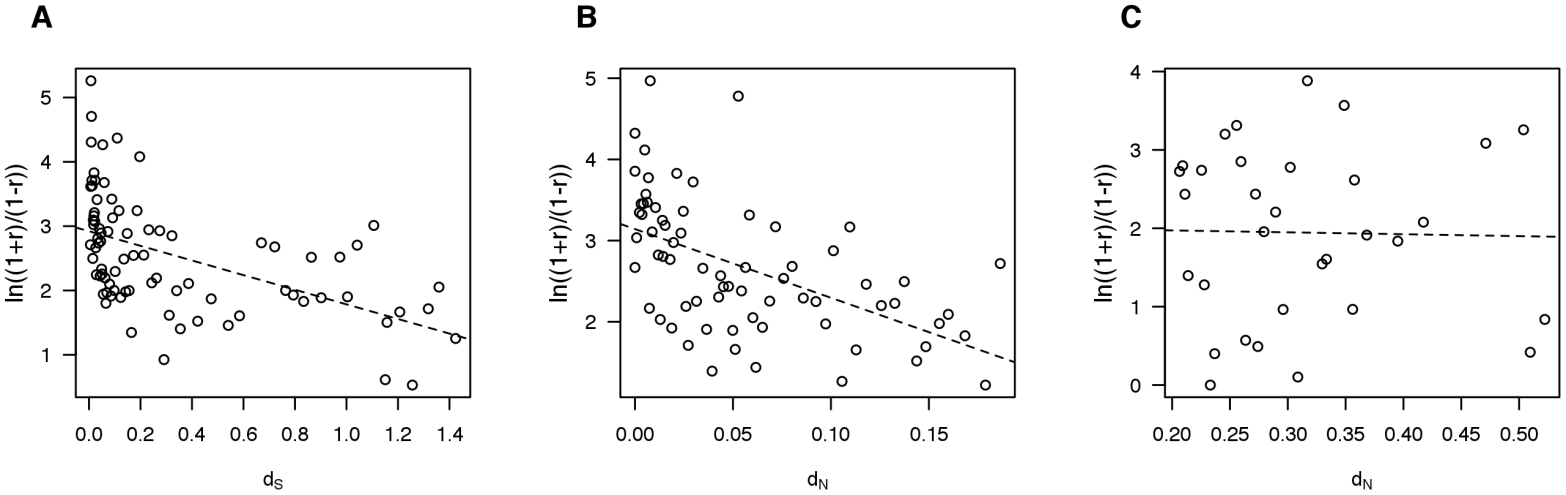
The relationship between Pearson’s correlation coefficient of gene expression and sequence divergence. (A) A significant negative correlation implies a positive correlation between *d_s_* and expression divergence because 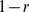 can be regarded as expression divergence. Each point represents five gene pairs. (B) A negative correlation between transformed r and *d_N_* with 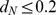. Each point represents five gene pairs. (C) No correlation between transformed r and *d_N_* with 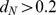. Each point represents five gene pairs.

To measure divergence in expression between duplicate genes, we performed transcriptome sequencing for the following tissues: adipose, muscle, hypothalamus, duodenum, liver, lung and kidney (library details are given in Table S1 and expression values are given in Table S4). We first calculated the proportion of duplicate genes with divergent expression. Two duplicate genes were treated as having diverged expression in a particular tissue if one gene is expressed in that tissue whereas the other is not. The expression data shows that 17–23% of duplicate gene pairs have divergence in one of the seven tissues studied (Table 2). In total, 59.8% of duplicate gene pairs have diverged in expression in at least one tissue and 38.6% of duplicate gene pairs have diverged in at least two tissues. Another way of measuring divergence in expression between duplicate genes is to compute Pearson’s correlation coefficient r. A significant negative correlation is observed between transformed r and *d_s_* (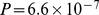, Figure 3A). In addition, a weaker negative correlation r is observed between *d_N_* and transformed r when only gene pairs with 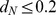 are examined (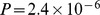, Figure 3B). With 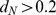, the correlation is no longer statistically significant (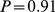, Figure 3C). Here, we analyzed the gene pairs in which both genes were expressed in at least one tissue. We observed the same significant relationships between expression divergence and sequence divergence when the analysis was restricted to pairs of genes that were expressed in three or more tissues (Figure S2). These findings regarding divergence in the spatial pattern are consistent with previous studies in yeast and human [10], [13].

**Table 2:** Percentage of differentially expressed duplicate gene pairs by tissue.

Tissues	Percentage of all duplicate gene pairs	Percentage of cattle-specific duplicate gene pairs
adipose	20.66	13.32
liver	20.60	11.23
duodenum	21.49	11.37
hypothalamus	17.15	11.51
lung	21.88	13.34
muscle	22.52	15.59
kidney	20.72	11.80

Among the cattle-specific gene duplicates, which in general have diverged much more recently than the duplicates as a whole, expression divergence is also observed. 11–13% of cattle-specific duplicates showed evidence of divergence of transcriptional levels between the duplicates (Table 2). We next focused on cattle-specific duplicate gene pairs showing dramatic changes in expression, perhaps signaling important functional divergence. We examined cattle-specific duplicate gene pairs with a Pearson’s correlation coefficient 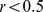. There were 31 such duplicate gene pairs in this group (Table S5). Interestingly, most of the gene pairs are structural divergent (30 of 31). These genes, which are enriched for certain functional activities, such as ‘helicase activity’ (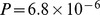), ‘hydrolase activity’ (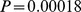), ‘catalytic activity’ (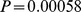), and ‘transferase activity’ (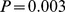), may have been targets of positive selection, perhaps through their contributions to important cattle adaptations.

### Relationship between structural divergence and expression divergence

**Figure 4:**
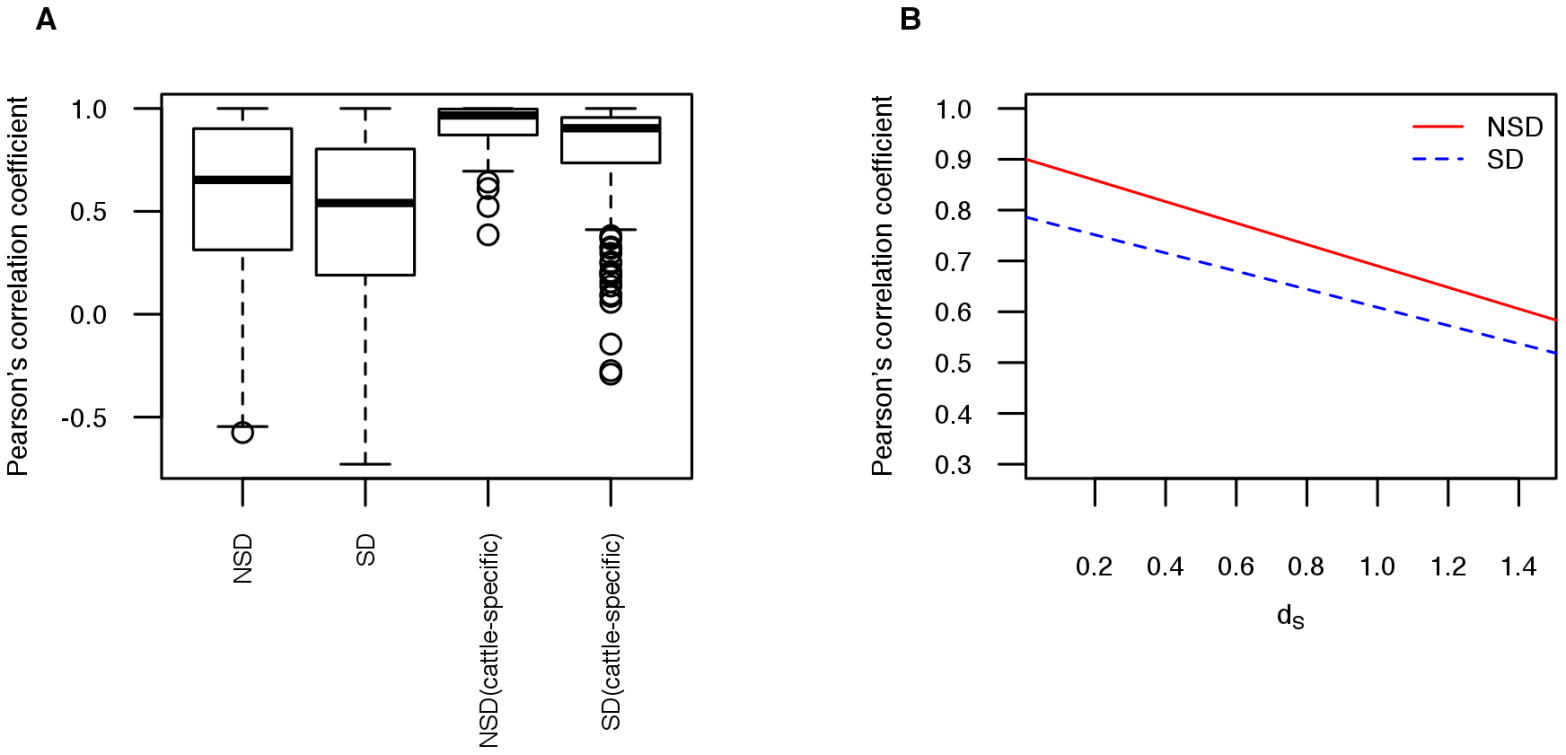
The relationship between Pearson’s correlation coefficient of gene expression and structural divergence. (A) Four different types of duplicate gene pairs: not structurally divergent (NSD), structurally divergent (SD), not structurally divergent cattle-specific (NSD cattle-specific), structurally divergent cattle-specific (SD cattle-specific). (B) The linear regression model with expression similarity as the dependent variable and the synonymous divergence as explanatory variable was constructed for both structurally divergent duplicate gene pairs and not structurally divergent duplicate gene pairs.

We compared the expression divergence of duplicate gene pairs with conserved gene structure to those with different exon-intron structure (Figure 4A). We observed that the expression divergence between structurally divergent duplicate gene pairs was significantly higher than the divergence between duplicate gene pairs with the same exon-intron structure, in comparisons involving all duplicate gene pairs (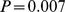) and in comparisons of cattle-specific duplicate gene pairs (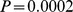). This result might be due to the fact that both structural and expression divergence are related with evolutionary time (synonymous divergence *d_s_*). We wondered if gene structure changes themselves account for some of the expression divergence, perhaps through altering, for example, transcription or splicing efficiency. We constructed a linear regression model with expression similarity as the dependent variable and the synonymous divergence as explanatory variable for both structurally divergent duplicate gene pairs and non-structurally divergent duplicate gene pairs. We found that expression divergence was on average greater for duplicate gene pairs exhibiting exon-intron structure divergence (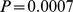), regardless of their overall level of synonymous sequence divergence (Figure 4B). Further analysis of covariance showed that a significant relationship exists between structural and expression divergences while controlling for the effect of synonymous sequence divergence (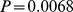). Restricting the analysis to genes expressed in three or more tissues did not change the findings (Figure S3).

## Discussion

### Cattle-specific duplicate gene pairs

Although gene duplications have been studied in the context of segmental duplications in cattle, we sought to more broadly characterize the repertoire and function of duplicate genes in this species using less stringent identity thresholds for discovery together with RNA-Seq for characterizing expression. Consistent with a previous cattle segmental duplication study [28], we observed many genes involved in ruminant or cattle specific aspects of reproduction including pregnancy-associated glycoprotein, interferon alpha and beta, trophoblast Kunitz domain proteins and prolactin-related proteins. These genes are related with fetal growth, maternal adaptations to pregnancy, and the coordination of parturition. We also found considerable gene duplications involved in adaptive immune responses in cattle. Duplication of genes involved in immune response or response to other organisms (e.g. bacterium) may be particularly important to cattle due to the substantial load of microorganisms present in the rumen [28]. We found that most duplicate gene pairs in cattle, including those classified as cattle-specific, exhibit structural divergence. Expression analysis led to the identification of a subset of cattle-specific duplicate gene pairs exhibiting high expression divergence. Because our analyses are based on currently available and undoubtedly imperfect genome assemblies and gene annotations, we expect that some of the duplicate pairs we identified and characterized are not bona fide gene pairs. Nonetheless, our results regarding cattle-specific duplicate genes should provide insight into the evolution of duplicate genes both at the sequence and expression levels.

### Relationship between structural divergence and expression divergence

Duplicate genes initially have similar sequences and functions but tend to divergence in regulatory and coding regions. It has been shown that the protein sequence divergence is positively correlated with expression divergence in *Drosophila* [43]. However, the relationship between exon-intron structure and expression divergence is not well understood. In a previous study of duplicate genes in *Arabidopsis thaliana* [8], structural divergence were found to be positively correlated with expression divergence. However, synonymous divergence was not taken into account in this previous study. In the present study, we found a significant relationship between structural and expression divergence while controlling the effect of synonymous sequence divergence. We can think of several explanations for this relationship. For example, positive selection could sometimes favor both gene structure and expression changes in the same gene because both might be contribute to a new beneficial function; genes subjected to relaxed negative selection may tend to accumulate both gene structure-altering and expression-altering mutations; and changes in gene structure might directly alter expression, though effects on transcription or transcripts.

## Conclusion

In this study, we identified duplicate genes in the bovine genome and further classified them as cattle-specific and non-cattle-specific. We found that structural divergence is common between duplicate genes and increases with evolutionary time, as expected. Using RNA-Seq, we investigated the relationship between gene expression and other characteristics and found a positive correlation between expression divergence and synonymous sequence divergence, as well as a significant relationship between structural and expression divergence while controlling the effect of evolutionary time. Our findings not only further support previously observed relationships observed in other species, but also describe a set of cattle-specific duplicate gene pairs, some of which may contribute to adaptations in this species.

## Supporting Information

Figure S1 **Histograms of Pearson’s correlation coefficient r and transformed r.** (A) Pearson’s correlation coefficient r. (B) The transformation (1+r)/(1–r). (C) The log transformation of (1+r)/(1–r).
(TIFF)

Figure S2 **The relationship between Pearson’s correlation coefficient of gene expression and sequence divergence when the analysis was restricted to pairs of genes that were expressed in three or more tissues.**
(TIFF)

Figure S3 **The relationship between Pearson’s correlation coefficient of gene expression and structural divergence when the analysis was restricted to pairs of genes that were expressed in three or more tissues.**
(TIFF)

Table S1 Details of sequencing libraries.
(XLSX)

Table S2 Complete list of duplicated gene pairs.
(XLSX)

Table S3 GO enrichment analysis of Cattle-specific duplicate genes.
(XLSX)

Table S4 Expression values in the seven tissues studied.
(XLSX)

Table S5 Cattle-specific duplicate genes that rapidly diverged in expression.
(XLSX)
