# Supplementary figures and images for: Sequence, Structural and Expression Divergence of Duplicate Genes in the Bovine Genome
Source: PLoS One. 2014 Jul 23;9(7):e102868. doi: 10.1371/journal.pone.0102868 (PMC4108385; doi:10.1371/journal.pone.0102868)

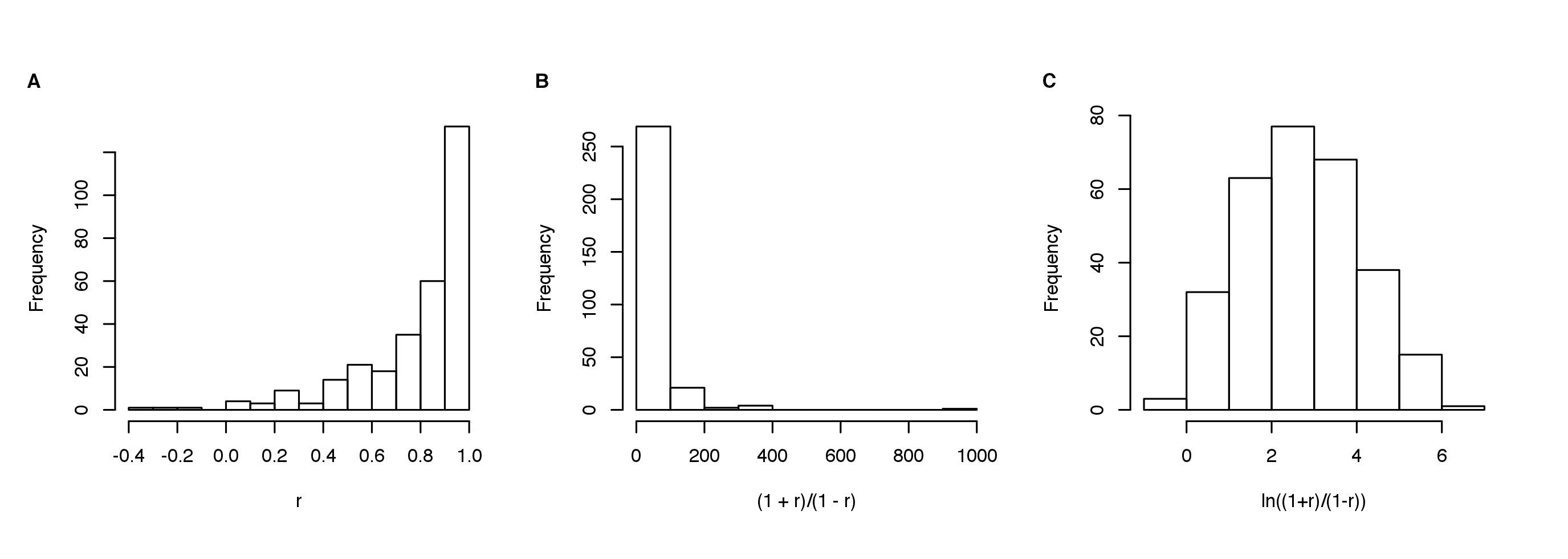

Supplement: Figure S1 — Histograms of Pearson’s correlation coefficient r and transformed r. (A) Pearson’s correlation coefficient r. (B) The transformation (1+r)/(1–r). (C) The log transformation of (1+r)/(1–r). (TIFF) [file pone.0102868.s001.tiff]

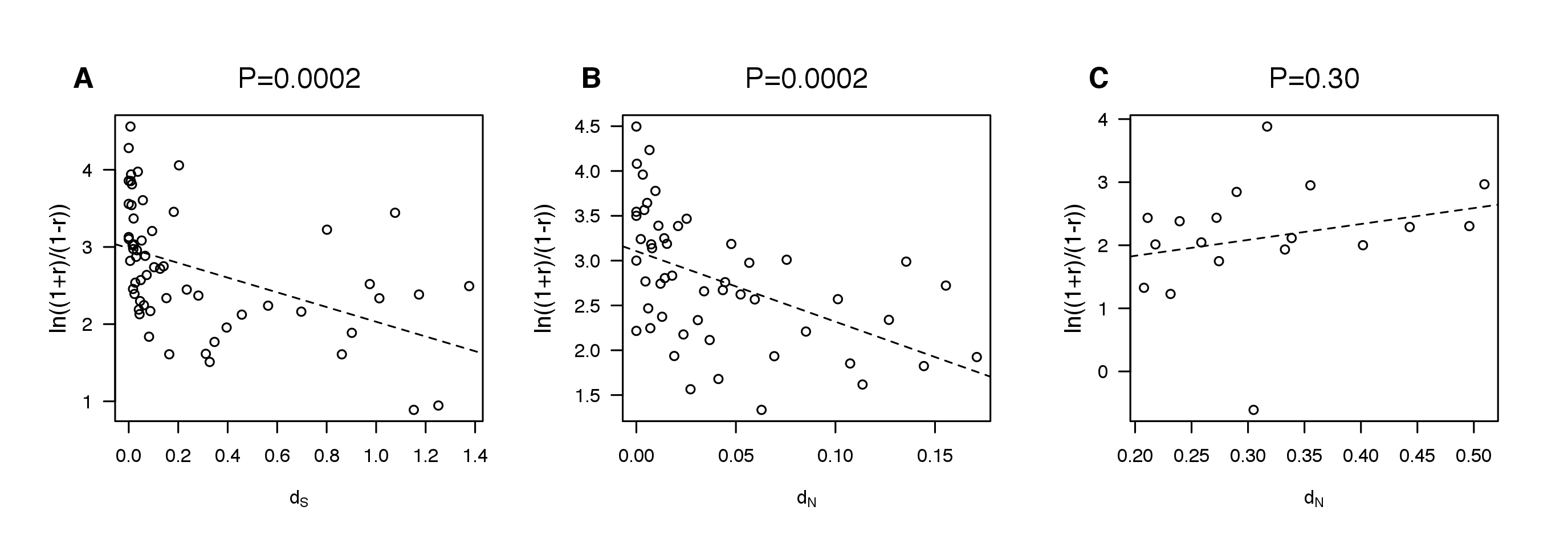

Supplement: Figure S2 — The relationship between Pearson’s correlation coefficient of gene expression and sequence divergence when the analysis was restricted to pairs of genes that were expressed in three or more tissues. (TIFF) [file pone.0102868.s002.tiff]

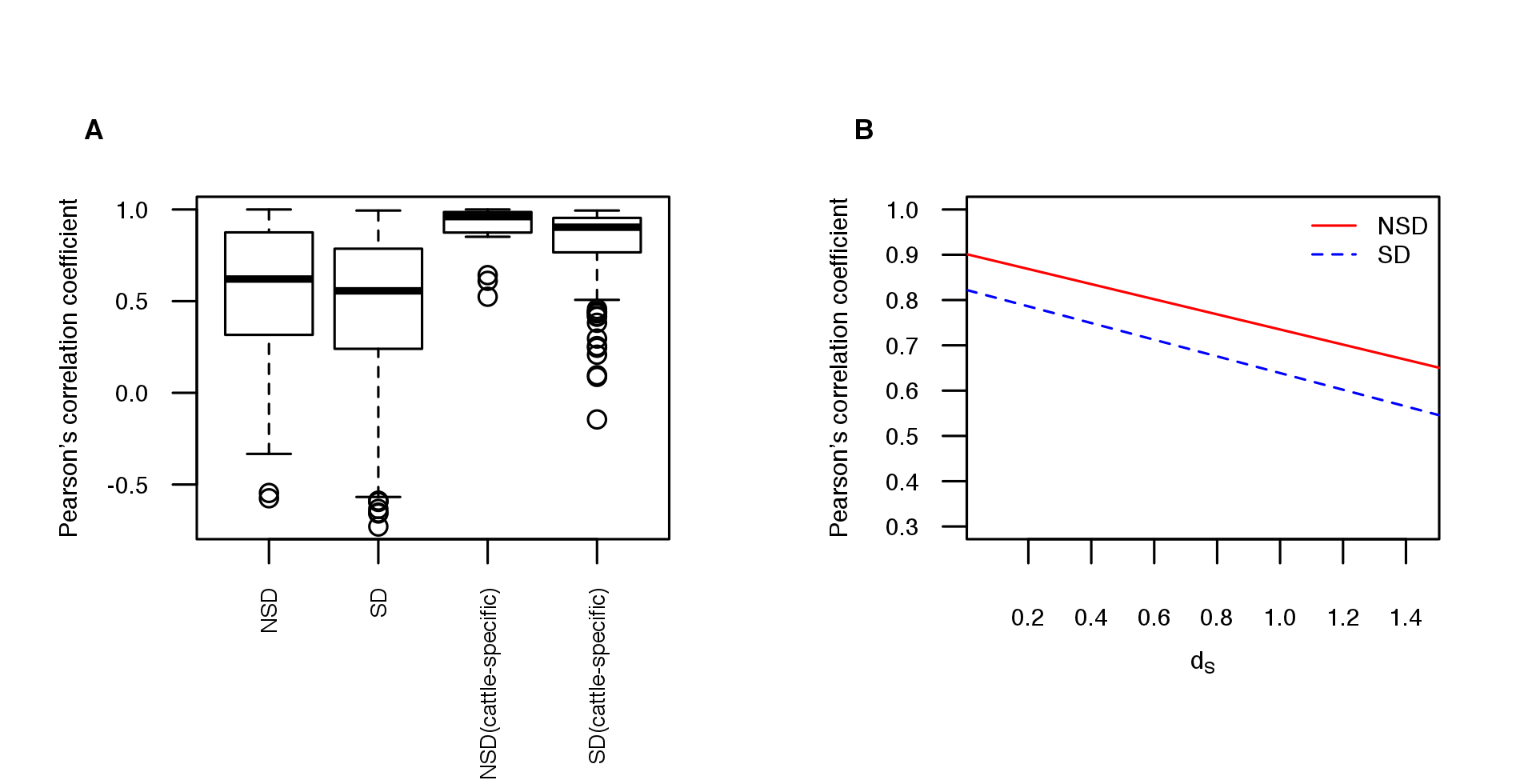

Supplement: Figure S3 — The relationship between Pearson’s correlation coefficient of gene expression and structural divergence when the analysis was restricted to pairs of genes that were expressed in three or more tissues. (TIFF) [file pone.0102868.s003.tiff]
